# Cordycepin Protects against Hepatic Ischemia/Reperfusion Injury via Inhibiting MAPK/NF-*κ*B Pathway

**DOI:** 10.1155/2022/5676256

**Published:** 2022-07-22

**Authors:** Jiameng Ding, WenjuanYang, Yuhui Jiang, Jie Ji, Jie Zhang, Liwei Wu, Jiao Feng, Yuanyuan Zheng, Yan Li, Ziqi Cheng, Qiang Yu, Jianye Wu, Jingjing Li, Kan Chen, Chuanyong Guo

**Affiliations:** ^1^Department of Gastroenterology, Shanghai Tenth People's Hospital, Tongji University School of Medicine, Shanghai 200072, China; ^2^Department of Emergency, Shanghai Tenth People's Hospital, School of Medicine, Shanghai 200072, China; ^3^Department of Gastroenterology, Putuo People's Hospital, Tongji University, Shanghai 200060, China

## Abstract

Hepatic ischemia/reperfusion injury (HIRI) is a common complication of liver surgery requiring hepatic disconnection, such as hepatectomy and liver transplantation. The aim of this study was to investigate the effects of cordycepin on HIRI and to elucidate the underlying mechanisms. Balb/c mice were randomly divided into six groups: a normal control group, sham group, H-cordycepin group, HIRI group, L-cordycepin (25 mg/kg) + HIRI group, and H-cordycepin (50 mg/kg) + HIRI group. Mice were subjected to I/R, and cordycepin was intragastrically administered for seven consecutive days before surgery. Orbital blood and liver specimens were collected at 6 and 24 h after HIRI. Serum levels of ALT and AST were decreased in the cordycepin pretreatment groups. Notably, cordycepin attenuated the inflammatory response and the production of proapoptosis proteins, while increasing expression of antiapoptosis proteins and decreasing expression of autophagy-linked proteins. Furthermore, cordycepin inhibited activation of the MAPK/NF-*κ*B signaling pathway. Collectively, these results indicate that cordycepin pretreatment ameliorated hepatocyte injury caused by HIRI. As compared with the HIRI group, cordycepin pretreatment mitigated the inflammatory response and inhibited apoptosis and autophagy via regulation of the MAPK/NF-*κ*B signaling pathway.

## 1. Introduction

Hepatic ischemia/reperfusion injury (HIRI) is a complex pathophysiological process of prolonged ischemia of hepatic tissue following reperfusion of blood flow after hepatectomy, liver transplantation, and trauma, leading to hepatocyte damage and organ dysfunction, seriously affecting the prognosis of patients [1]. HIRI involves multiple mechanisms, which has been shown to affect the functions of many remote organs, including the lung, kidney, and myocardium [2]. The initial stage of reperfusion is the onset of inflammation, which is characterized by the activation of Kupffer cells, following the production and release of inflammatory cytokines and reactive oxygen species [3]. The acute inflammatory response produces various inflammatory factors, including tumor necrosis factor- (TNF-) *α*, interleukin- (IL-) 1, and IL-6, which damage the liver [4]. At present, there is no effective treatment for liver ischemia-reperfusion injury in clinic. Therefore, exploring the potential pathophysiological mechanisms of HIRI and finding effective treatment measures are the current problems to be solved.

Cordycepin is a metabolite produced by the fungus Cordyceps militaris with antivirus activities and conveys a variety of pharmacological functions, including immunomodulation, neuroprotection, anti-inflammation activities, and even effect tumorigenesis and cancer progression [5–8]. Cordycepin ameliorates inflammation by inhibiting the release of proinflammatory cytokines, such as IL-1*β* and IL-6, and exhibits anti-inflammatory effects via Toll-like receptor 4-mediated inhibition of the mitogen-activated protein kinase (MAPK) and nuclear factor- (NF-) *κ*B signaling pathways [9]. Many studies have shown anti-inflammatory and antiapoptotic effects of cordycepin of attenuate I/R injury in the brain, kidney, and heart [10–12]. However, there is no literature report on the protective effect of cordycepin on HIRI, and the effect of cordycepin on HIRI remains unclear.

Apoptosis, also known as type I programmed death, is a process of active cell death under gene regulation during ontogenesis. Activation of the apoptotic machinery is divided into an intrinsic mitochondria-dependent mediated pathway and an extrinsic death receptor-dependent triggered pathway [13, 14]. Apoptosis is reportedly involved in the pathogenesis of acute liver injury, hepatitis, and liver cancer [15–17]. During reperfusion, TNF-*α* and other mediators activate many proteins involved in apoptosis, such as the protease caspase-3, caspase-9, and bax [18, 19]. Autophagy, also known as type II programmed cell death, is a lysosomal degradation pathway that can isolate damaged, excess, or aging cytoplasmic components and plays an important role in cell homeostasis and regulation of the intercellular environment [20, 21]. Autophagy is a bidirectional regulatory process that is activated under extremely stressful conditions, resulting in hepatocyte apoptosis and necrosis [22]. IR is a multifactorial process; the role of autophagy in IR injury has been extensively studied; autophagy-related proteins, such as Beclin1 and P62, are significantly upregulated during ischemia-reperfusion [23, 24].

Ray and Sturgill first characterized a novel serine/threonine kinase in animal cells known as MAPK [25, 26]. The MAPK signaling pathway regulates various cellular mechanisms, including inflammation, apoptosis, and autophagy [27, 28]. NF-*κ*B is a key transcriptional regulator of the inflammatory response [29]. Activation of this signaling pathway is crucial to cell death, inflammation, and wound healing and is an important regulator of the progression of liver diseases [30]. MAPK is an upstream activator and regulator of the NF-*κ*B pathway [31]. Hence, the relationship between MAPK and NF-*κ*B is worth investigating.

Therefore, the aims of the present study were to determine whether cordycepin is protective against HIRI and to elucidate the underlying mechanism. We hypothesized that cordycepin preconditioning could diminish the inflammatory response, apoptosis, and autophagy by partially inhibiting activation of the MAPK/NF-*κ*B signaling pathway.

## 2. Materials and Methods

### 2.1. Study Approval

All animal experiments were approved by the Institutional Animal Care and Use Committee of Tongji University (Shanghai, China) and conducted in accordance with the Guide for the Care and Use of Laboratory Animals (National Institutes of Health, Bethesda, MD, USA).

### 2.2. Drugs and Reagents

Cordycepin was purchased from Shanghai Yuanye Bio-Technology Co., Ltd. (Shanghai, China) and suspended in double distilled H20. Alanine aminotransferase (ALT) and aspartate aminotransferase (AST) microplate test kits were purchased from Nanjing Jiancheng Bioengineering Institute (Nanjing, China). Quantitative real-time polymerase chain reaction (qRT-PCR) kits were obtained from Takara Bio, Inc. (Shiga, Japan). A terminal deoxynucleotidyl transferase dUTP nick end labeling (TUNEL) assay kit was obtained from Hoffmann-La Roche AG (Basel, Switzerland).

### 2.3. Animals

Healthy male BALB/c mice (*n* = 60; body mass, 23 ± 2 g; age, 6–8 weeks) were purchased from Shanghai SLAC Laboratory Animal Co., Ltd. (Shanghai, China) and housed in sterilized cages at a constant temperature of 24 ± 2°C under a 12 h light/dark cycle with ad libitum access to feed and water.

### 2.4. Establishment of the HIRI Model

Mice were subjected to segmental (70%) hepatic warm ischemia to induce HIRI [18]. Before surgery, the mice were fasted for 24 h with access to water only and then anesthetized by intraperitoneal injection of 1.25% sodium pentobarbital (nembutal sodium; Sigma-Aldrich Corporation, St. Louis, MO, USA). After the mice no longer responded to painful stimuli, an incision was made along the abdominal white line. Then, the hilar structure was carefully isolated, and the left and middle lobe vessels of the liver were blocked with sterile microarterial clamps, which instantly caused liver partial warm ischemia. The abdomen was covered with saline gauze, and the mice were warmed with electric blankets. After 45 min, the arteriole clamp was removed to reestablish blood flow and reperfusion, the abdominal incision was closed layer by layer, and the mice were warmed on an electric blanket until revived from the anesthesia.

### 2.5. Experimental Design

The 60 mice were randomly assigned to one of six groups: (1) a normal control (NC) group (*n* = 6; untreated), (2) H-cordycepin (50 mg/kg) group (*n* = 6; pretreatment with cordycepin at 50 mg/kg once daily for 7 days without HIRI surgery), (3) sham surgery group (*n* = 12; the abdominal cavity was opened to separate the first hepatic portal and quickly closed, without HIRI surgery), (4) IR group (*n* = 12; HIRI without cordycepin pretreatment), (5) HIRI + L-cordycepin group (*n* = 12; pretreatment with cordycepin at 25 mg/kg once daily for 7 days before surgery), or (6) HIRI + H-cordycepin group (*n* = 12; pretreatment with cordycepin at 50 mg/kg once daily for 7 days before surgery).

Six mice were randomly selected from each group at 6 and 24 h after initiation of reperfusion, and blood and liver tissues were collected for further biochemical analysis.

### 2.6. Serum Biochemical Analysis

The blood samples were cooled for 5 h at 4°C, then centrifuged at 4600 rpm for 10 min, and the supernatant was stored at −80°C until analyzed for serum ALT and AST levels.

### 2.7. Histopathological Analysis

A portion of the liver lobe was fixed in 4% paraformaldehyde for 24 h, then dehydrated with ethanol, embedded in paraffin, and cut into 5 *μ*m-thick sections, which were stained with hematoxylin and eosin (H&E) to observe the severity of liver injury under an optical microscope.

### 2.8. Immunohistochemical Analysis

The paraffin-embedded sections were preheated for 2 h to 60°C, immersed in xylene for 30 min, then dehydrated with gradient alcohol, and placed in sodium citrate repair solution for 10 min at 95°C. Afterward, the slices were incubated with 3% hydrogen peroxide for 10 min at room temperature in the dark to block endogenous peroxidase activity. After washing three times with phosphate-buffered saline (PBS), 5% bovine serum albumin was added to block nonspecific proteins, and the slices were probed with antibodies against TNF-*α*, IL-6, IL-1*β*, Beclin-1, microtubule-associated protein 1A/1B-light chain 3 (LC3), Bax, Bcl-2, caspase-3, caspase-9, NF-*κ*B, and phosphorylated MAPK (p-MAPK) overnight at 4°C followed by a secondary antibody (dilution, 1 : 50) for 1 h at 37°C. Finally, a DAB kit was used to analyze the bound antibodies under a light microscope. The Image-Pro Plus software (version 6.0) was used to measure the integrated optical densities (IOD) of sections and analyze differences.

### 2.9. Western Blot Analysis

The total protein contents of liver tissues preserved in liquid nitrogen were extracted with radioimmunoprecipitation assay lysis buffer containing protease inhibitors and phenylmethylsulfonyl fluoride, mixed with 5× loading buffer, and boiled for 10 min at 100°C. Then, the protein concentrations were determined with a bicinchoninic acid protein assay kit (Kelley, China). The protein samples were loaded into the wells of 10% and 12.5% dodecyl sulfate polyacrylamide gels, separated by electrophoresis, and then transferred onto methanol-activated polyvinylidene fluoride membranes, which were blocked at room temperature with PBS containing 5% nonfat milk for at least 1 h and then probed with antibodies against TNF-*α* (dilution, 1 : 500), IL-1*β* (1 : 1000), IL-6 (1 : 1000), Beclin-1 (1 : 1000), LC3 (1 : 1000), P62 (1 : 1000), Bcl-2 (1 : 1000), Bax (1: 1000), caspase-3 (1 : 1000), caspase-9 (1 : 1000), MAPK (1 : 1000), p-MAPK (1 : 1000), NF-*κ*B (1 : 1000), and *β*-actin (1 : 1000) overnight at 4°C. The next day, the membranes were washed three times for 10 min with PBS containing 0.1% Tween 20 (PBST), then incubated with secondary antibodies at 1 : 2000 for 1 h, washed three times with PBST, and scanned with an Odyssey two-color infrared laser imaging system (LI-COR Biosciences, Lincoln, NE, USA), and the gray values were quantified using the ImageJ v1.8.0 software.

### 2.10. RNA Isolation and qRT-PCR

Total RNA was extracted from the liver tissue samples using TRIzol reagent and then reverse transcribed into complementary DNA with the use of a commercial kit (Takara Bio, Inc.) and SYBR Premix Ex Taq polymerase (Takara Bio, Inc.) with the primers listed in Table 1. The resulting cDNA was quantified using a 7900HT Fast Real-Time PCR System (Applied Biosystems, Carlsbad, CA, USA).

### 2.11. TUNEL Staining

Apoptosis of hepatocytes was detected with the TUNEL assay. The prepared slices were dewaxed, dehydrated, and then digested with 20 *μ*g/mL of protease K. After rinsing four times, the slices were treated with TUNEL reaction buffer. Finally, the positive regions were observed under a light microscope. The ImageJ v1.8.0 software was used to observe and analyze TUNEL results.

### 2.12. Statistical Analysis

All experimental data are expressed as the mean ± standard deviation. One-way analysis of variance was performed for comparisons of the data among the groups. A probability (*p*) value < 0.05 was considered statistically significant. Figures were created using the GraphPad Prism software (v9.0; GraphPad Software, Inc., San Diego, CA, USA).

## 3. Results

### 3.1. Cordycepin Had No Negative Influence on Normal Liver Function and Structure

**Figure 1:**
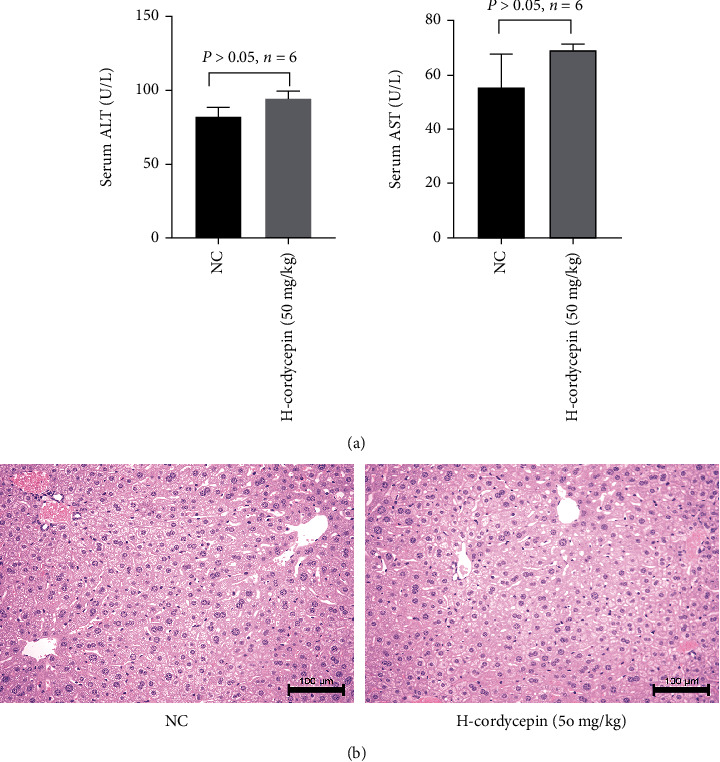
Cordycepin had no side effects on liver structure and function. Note: (a) the levels of ALT and AST were shown as mean ± SD (*n* = 6; *p* > 0.05). It indicated that there was no significant difference among the two groups. (b) Representative H&E-stained hepatic sections were examined under light microscopy and imaged at a ×200 magnification.

The preliminary experimental results showed that there was no significant difference in serum ALT and AST levels between the H-cordycepin and NC groups, indicating that liver function was normal (Figure 1(a)). In addition, there was no significant necrosis of hepatocytes in the H&E-stained sections of either group (Figure 1(b)). The results showed that high concentrations of cordycepin (50 mg/kg) did not cause liver tissue damage, indicating that the concentrations used in this study were safe and reliable.

### 3.2. Cordycepin Preconditioning Alleviated HIRI

**Figure 2:**
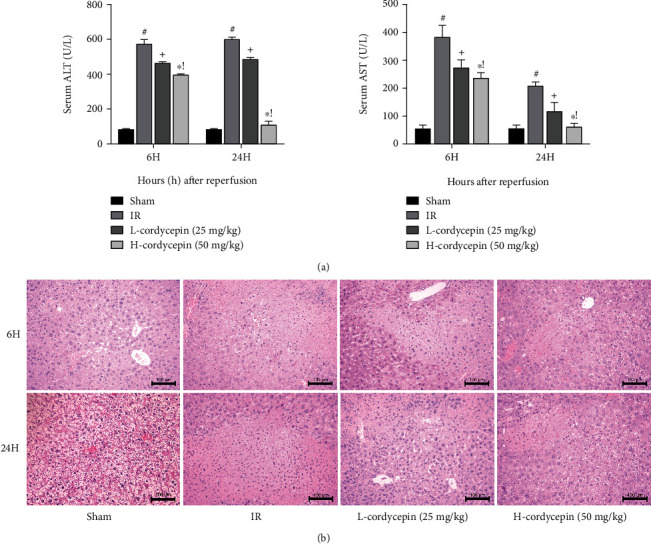
Cordycepin pretreatment could significantly alleviate liver injury after ischemia-reperfusion. Note: (a) the levels of serum ALT and AST were presented as mean ± SD (*n* = 6; #*p* < 0.05 for IR vs. sham; +*p* < 0.05 for L-cordycepin (25 mg/kg) vs. IR; ^∗^*p* < 0.05 for H-cordycepin (50 mg/kg) vs. IR; !*p* < 0.05 for L-cordycepin (25 mg/kg) vs. H-cordycepin (50 mg/kg)). (b) Representative H&E-stained hepatic sections were examined under light microscopy and imaged at ×200 magnification.

As biomarkers of liver function, serum ALT and AST levels reflect the degree of acute liver injury. As shown in Figure 2, serum ALT and AST levels were significantly higher in the HIRI group than the sham group, indicating that the HIRI model was successfully established. Besides, ALT and AST levels were significantly reduced in the cordycepin pretreatment groups with a greater decrease in the high-dose group than the low-dose group (Figure 2(a)). These results indicate that cordycepin reduced the contents of liver enzymes in a dose-dependent manner. Also, H&E staining of the liver specimens was performed to further verify the influence of cordycepin pretreatment on HIRI in terms of pathological structure. No necrosis was observed in the sham group, but necrosis and extensive infiltration of inflammatory cells were seen in the HIRI group. As compared with the HIRI group, cordycepin pretreatment significantly reduced hepatocyte necrosis, especially in the high-dose group (50 mg/kg) (Figure 2(b)). Collectively, these findings confirmed that cordycepin pretreatment alleviated HIRI-induced liver damage.

### 3.3. Cordycepin Reduced HIRI-Induced Systemic Inflammation

**Figure 3:**
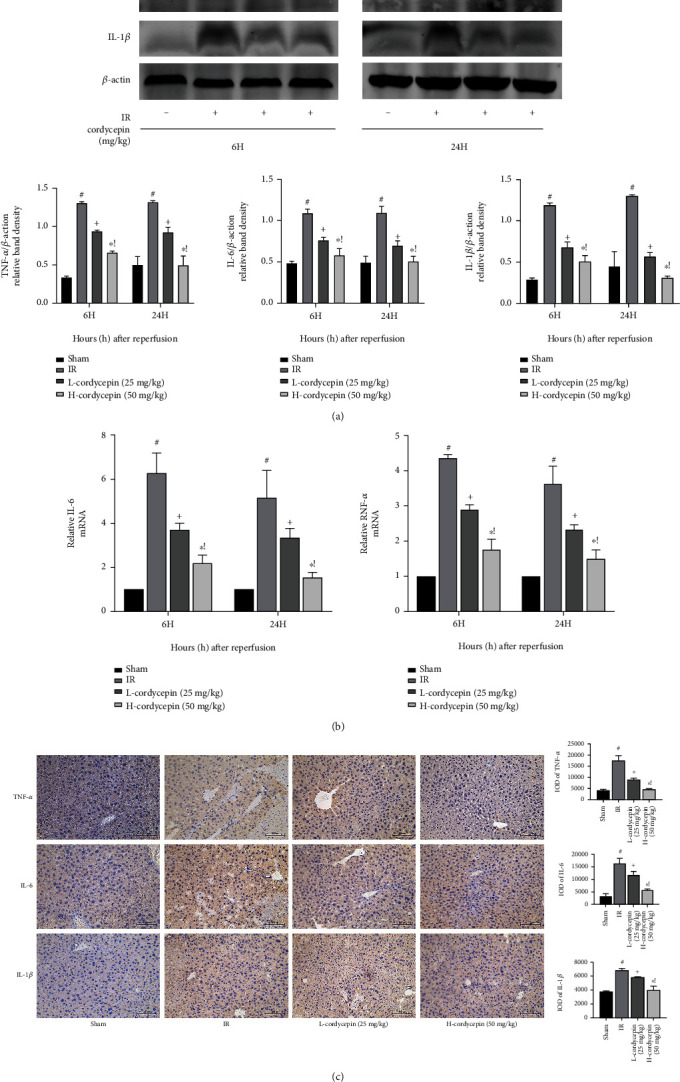
Cordycepin reduced systemic inflammatory responses induced by hepatic IR. Note: (a) western blot analysis of TNF-*α*, IL-6, and IL-1*β* protein levels. (b) Relative TNF-*α*, IL-6, and IL-1*β* levels were determined by qRT-PCR. (c) TNF-*α*, IL-6, and IL-1*β* protein expressions in liver tissues after reperfusion are shown by immunohistochemical staining (×200). The evaluations were made by the Image-Pro Plus 6.0 software to calculate the IOD of the positive staining area. Data are presented as mean ± SD (*n* = 6; #*p* < 0.05 for IR vs. sham; +*p* < 0.05 for L-cordycepin (25 mg/kg) vs. IR; ^∗^*p* < 0.05 for H-cordycepin (50 mg/kg) vs. IR; !*p* < 0.05 for L-cordycepin (25 mg/kg) vs. H-cordycepin (50 mg/kg)).

The release of proinflammatory cytokines is an important characteristic of HIRI. Therefore, in this experiment, expression levels of TNF-*α*, IL-6, and IL-1*β* were assessed by western blot, qRT-PCR, and immunohistochemical analyses. The western blot results showed that the expression levels of the proinflammatory cytokines were significantly higher in the HIRI group than the sham group at both timepoints (6 and 24 h), while comparatively reduced in the cordycepin pretreatment group, especially the high-dose group (50 mg/kg) (Figure 3(a)). This phenomenon was further verified by qRT-PCR and immunohistochemical analyses (Figures 3(b) and 3(c)). These findings confirmed that cordycepin attenuated inflammation during HIRI.

### 3.4. Cordycepin Inhibited HIRI-Associated Hepatocyte Apoptosis and Autophagy

**Figure 4:**
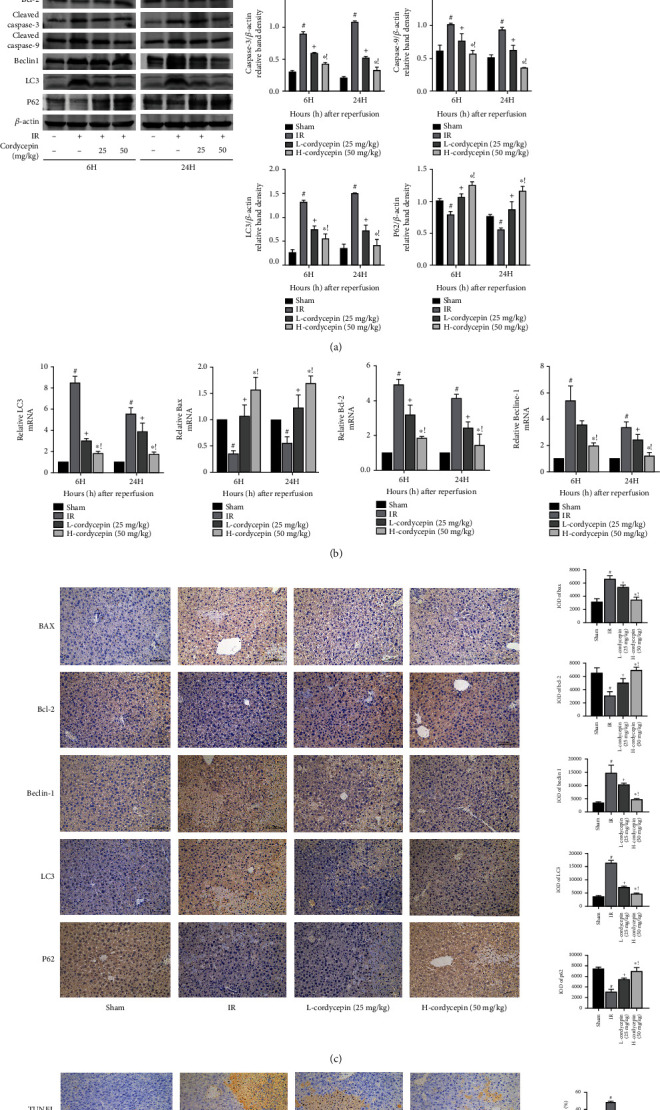
Cordycepin can reduce hepatocyte apoptosis and autophagy induced by HIRI. Note: (a) western blot analysis of Bax, Bcl-2, caspase-3, caspase-9, Beclin-1, LC3, and P62 protein levels. (b) Relative Bax, Bcl-2, Beclin-1, and LC3 levels were determined by qRT-PCR. (c) Bax, Bcl-2, Beclin-1, LC3, and P62 protein expressions in liver tissues after reperfusion are shown by immunohistochemical staining (×200). The evaluations were made by the Image-Pro Plus 6.0 software to calculate the IOD of the positive staining area. (d) TUNEL staining showed apoptotic hepatocytes in four groups at 6 h. Final evaluations were made using the ImageJ v1.8.0 software to calculate positive rate. Data are presented as mean ± SD (*n* = 6; #*p* < 0.05 for IR vs. sham; +*p* < 0.05 for L-cordycepin (25 mg/kg) vs. IR; ^∗^*p* < 0.05 for H-cordycepin (50 mg/kg) vs. IR; !*p* < 0.05 for L-cordycepin (25 mg/kg) vs. H-cordycepin (50 mg/kg)).

The pathological process of HIRI also includes hepatocyte apoptosis and autophagy. Caspase-3, caspase-9, and Bax were proapoptotic proteins, while Bcl-2 has an opposite effect. First, western blot was used to detect related proteins, and the results showed that expression of the antiapoptotic protein Bcl-2 was relatively low in the HIRI group but significantly increased in the cordycepin pretreatment groups. Meanwhile, the expression levels of the proapoptotic proteins Bax, caspase-3, and caspase-9 were elevated in the HIRI group but decreased in the cordycepin pretreatment groups (Figure 4(a)). The western blot and qRT-PCR results showed similar trends (Figure 4(b)). The immunohistochemical results are shown in Figure 4(c). Western blot and qRT-PCR analyses found that the expression levels of the autophagy-related proteins Beclin-1 and LC3 were upregulated, while expression of the antiautophagy protein P62 was downregulated, as compared with the sham group (Figures 4(a) and 4(b)). After cordycepin pretreatment, the expression levels of Beclin-1 and LC3 were decreased, while that of P62 was increased. Finally, TUNEL staining to assess the degree of apoptosis in liver tissue (Figure 4(d)) found comparatively high numbers of apoptotic hepatocytes in the HIRI groups, while the proportion of TUNEL-positive cells was significantly reduced in the cordycepin-pretreatment groups. Together, these results confirmed that cordycepin reduced apoptosis and autophagy during HIRI in mice.

### 3.5. The Protective Effect of Cordycepin against HIRI Is Related to the MAPK/NF-*κ*B Signaling Pathway

**Figure 5:**
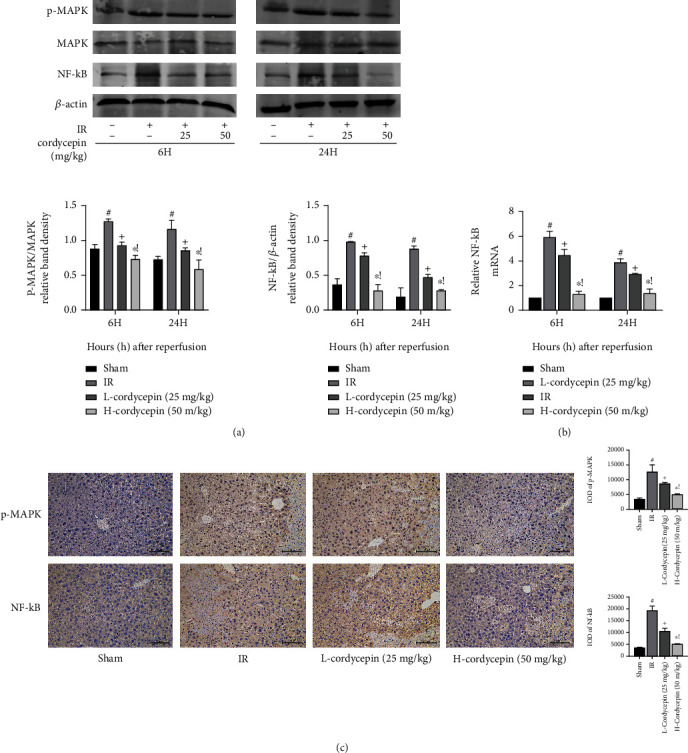
The protective effect of cordycepin on HIRI in mice was related to MAPK/NF-*κ*B signaling pathway. Note: (a) western blot analysis of MAPK, p-MAPK, and NF-*κ*B protein levels. (b) Relative NF-*κ*B levels were determined by qRT-PCR. (c) p-MAPK and NF-*κ*B protein expressions in liver tissues after reperfusion are shown by immunohistochemical staining (×200). The evaluations were made by the Image-Pro Plus 6.0 software to calculate the IOD of the positive staining area. Data are presented as mean ± SD (*n* = 6; #*p* < 0.05 for IR vs. sham; +*p* < 0.05 for L-cordycepin (25 mg/kg) vs. IR; ^∗^*p* < 0.05 for H-cordycepin (50 mg/kg) vs. IR; !*p* < 0.05 for L-cordycepin (25 mg/kg) vs. H-cordycepin (50 mg/kg)).

The MAPK/NF-*κ*B signaling pathway also plays an important role in regulating the inflammatory response and apoptosis. We adopted western blot, qRT-PCR, and IHC to detect the expression of MAPK and NF-*κ*B. The experimental results showed that cordycepin had no effect on MAPK expression. However, there were significant differences in p-MAPK expression levels between the HIRI and IR + cordycepin (25 and 50 mg/kg) groups. The relative lower expression of p-MAPK in the IR + cordycepin groups indicated that cordycepin significantly inhibited phosphorylation of MAPK. In addition, NF-*κ*B was significantly downregulated in the cordycepin-pretreatment groups, which was more obvious with an increased dosage (Figure 5(a)). This phenomenon was further confirmed by qRT-PCR and immunohistochemical analyses (Figures 5(b) and 5(c)). These results suggest that the protective effect of cordycepin on HIRI is partly related to inhibition of the MAPK/NF-*κ*B signaling pathway.

## 4. Discussion

HIRI is commonly caused by surgical procedures, such as hepatectomy and liver transplantation [32]. HIRI is a pathophysiological process that is further exacerbated during reperfusion after a period of hepatic ischemia. After reperfusion, the onset of an acute inflammatory response is a key factor leading to tissue damage and associated with apoptosis, autophagy, and oxidative stress [23]. This disease can seriously affect the prognosis of patients, resulting in liver cell damage and organ dysfunction. At present, there is no effective treatment for HIRI. Therefore, the underlying mechanisms should be explored to develop potential strategies for the prevention and treatment of HIRI. Cordycepin is a natural active substance mainly extracted from C. militaris with anti-inflammatory, antioxidant, antitumor, antimetastasis, and immunomodulatory effects [33–35]. However, it remains unclear whether cordycepin has hepatoprotective effects. Hence, a mouse model of HIRI was created to determine whether cordycepin can protect against HIRI and to elucidate the underlying mechanism.

Serum levels of certain enzymes like AST and ALT are one of the sensitive indicators established markers of the extent of liver damage [36]. In the present study, ALT and AST were significantly elevated in serum at two timepoints, indicating severe liver injury after reperfusion. Cordycepin pretreatment reduced liver enzyme activity in a dose-dependent manner. This conclusion was further validated by the pathological changes, which showed that cordycepin pretreatment reduced the extent of necrosis and inflammation as compared with the HIRI group, confirming the protective effect of cordycepin against HIRI in mice. The early stage of liver injury is characterized by the recruitment of Kupffer cells and neutrophils to sites of HIRI injury and the production of large amounts of reactive oxygen species, which induce oxidative stress, resulting in apoptosis and tissue damage [37]. Aggregates of neutrophils and T cells activated by Kupffer cells release a variety of proinflammatory cytokines, such as TNF-*α*, IL-6, and IL-1*β*, which aggravate HIRI. TNF-*α* is an inflammatory cytokine mainly secreted by activated monocytes and macrophages and plays a central role in triggering inflammatory responses [38]. In the present study, western blot, qRT-PCR, and immunohistochemical analyses were performed to detect levels of inflammatory factors in liver tissue. The results showed that cordycepin reduced expression of TNF-*α*, L-1*β*, and IL-6 in a dose-dependent manner, indicating that cordycepin can protect the liver by reducing the release of inflammatory mediators.

Apoptosis is the process of cell death that can occur through intrinsic or extrinsic pathways to maintain tissue homeostasis. Apoptosis of hepatocytes is mainly regulated by members of the Bcl-2 family. The upregulation of Bax to Bcl-2 ratio results in the release of cytochrome C in the early stage of apoptosis, leading to activation of caspase-9 [39]. Apoptosis is also orchestrated by members of caspase family, as the initiator caspase, caspase-9 cleaves and activates caspase-3 after activation [40]. Upregulation of Bcl-2 expression reduces hepatocyte apoptosis in liver diseases [41, 42]. In the present study, the expression of apoptosis-related proteins was examined, which showed that cordycepin pretreatment upregulated expression of Bcl-2 but downregulated expression of Bax. Caspase-9 and caspase-3 have similar trends to Bax. From the results of TUNEL staining, we found that the proportion of TUNEL-positive cells was significantly reduced in the cordycepin-pretreatment groups. Autophagy is involved in HIRI. Beclin-1, LC3, and P62 are biomarkers of autophagy. Beclin-1 interacts with Bcl-2 through the functional BH3 domain, which is located in the active sites of members of the antiapoptotic Bcl-2 family. The combination of Beclin-1 and Bcl-2 reduces the conversion of LC3 I to LC3 II, further inhibiting autophagy [43, 44]. P62 plays a key role in autophagy by binding to the LC3-interacting region [45]. We measured the signature proteins involved in autophagy by western blot, qRT-PCR, and HIC; it showed that after cordycepin pretreatment, the expression levels of Beclin-1 and LC3 were decreased, while that of P62 was increased. These findings demonstrate that cordycepin preconditioning can reduce liver injury by regulating the expression levels of proteins associated with apoptosis and autophagy.

As an important transcription factor, NF-*κ*B is involved in the expression and regulation of many genes closely related to various pathophysiological processes, such as inflammation, the immune response, and apoptosis [46]. The tumor necrosis factor receptor and Toll-like receptor/interleukin-1 receptor superfamilies can induce activation of NF-*κ*B during ischemia, with a significant increase in NF-*κ*B activity during the reperfusion phase [47, 48]. In addition, NF-*κ*B also induces the production of proinflammatory cytokines such as TNF-*α* and IL-1*β*, leading to inflammatory responses [49]. It has been reported that NF-*κ*B is associated with the inflammatory response, apoptosis, and autophagy during HIRI [24, 50–52]. Yang et al. studied that cordycepin can inhibit NF-*κ*B and NLRP3 inflammasome activation and reduce the inflammatory process to alleviate acute pancreatitis [53]. Thus, we detected the levels of NF-*κ*B protein and mRNA in liver tissue in this study. The results showed that NF-*κ*B expression was increased in HIRI, while cordycepin pretreatment inhibited NF-*κ*B expression.

MAPK is a class of intracellular serine/threonine protein kinases that can be activated by proinflammatory factors, including TNF-*α* and IL-1*β*, and in response to cellular stressors, such as genotoxicity, osmotic pressure, hypoxia, and oxidative stress [54]. As signal transmitters, MAPK-associated signaling pathways can convert extracellular stimuli into a variety of cellular responses [27]. Inactive MAPK is nonphosphorylated, while phosphorylated (activated) MAPK participates in regulation of the inflammatory response; promotes the release of cytokines, such as TNF-*α*, IL-1, IL-6, and IL-8; and activates the downstream transcription factor NF-*κ*B [55]. Li et al. found that death-associated protein kinase 1 ameliorates inflammation, oxidative stress, and autophagy in acute lung injury by inhibiting activation of the MAPK/NF-*κ*B signaling pathway [56]. Many studies have demonstrated that the MAPK signaling pathway is associated with liver fibrosis, nonalcoholic fatty liver disease, and autoimmune hepatitis [57–60]. In this study, qRT-PCR was employed to determine the expression profiles of p-MAPK and NF-*κ*B in HIRI, while western blot and immunohistochemical analyses were used to determine whether MAPK/NF-*κ*B plays a role in HIRI. The results showed that there was no significant difference in MAPK expression between the HIRI group and cordycepin pretreatment groups, while p-MAPK was significantly downregulated and NF-*κ*B expression was decreased. These findings demonstrated that cordycepin inhibited MAPK phosphorylation and inhibited NF-*κ*B activation, thereby reducing the release of proinflammatory factors.

**Figure 6:**
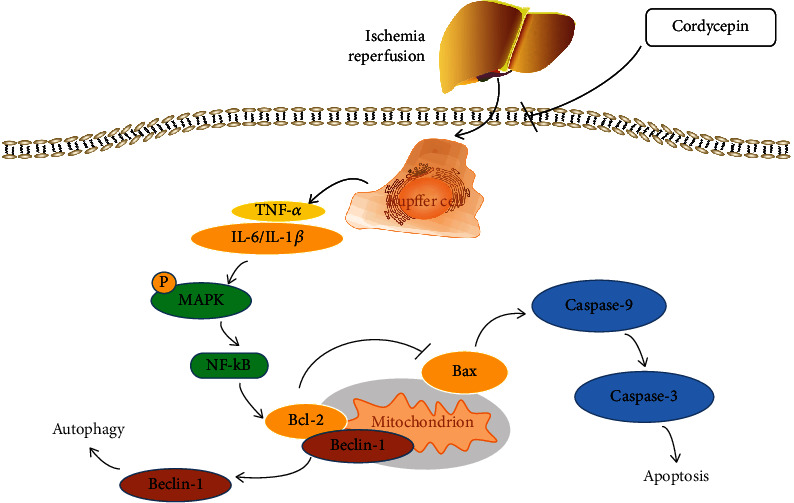
Possible mechanism of cordycepin in hepatic ischemia-reperfusion injury. In IR model, activated Kupffer cells promote the release of proinflammatory cytokines such as TNF-*α*, IL-1*β*, and IL-6, and cordycepin reduces inflammatory cytokines. In addition, cordycepin inhibited the phosphorylation of MAPK, which then reduce the expression of NF-*κ*B to effect hepatocyte apoptosis and autophagy. Cordycepin has a protective effect on ischemia-reperfusion liver by inhibiting inflammation and attenuating apoptosis and autophagy through the MAPK/NF-*κ*B pathway.

The results of this study suggest that cordycepin can regulate inflammation, apoptosis, and autophagy by inhibiting the MAPK/NF-*κ*B signaling pathway (Figure 6) and has a certain protective effect against HIRI, suggesting that cordycepin pretreatment has potential for treatment of HIRI. However, there were several limitations to this study. Notably, the relationship between cordycepin and the MAPK/NF-*κ*B signaling pathway remains unclear, and the mechanisms underlying the onset of HIRI are complex; thus, further studies are warranted.

## 5. Conclusions

Cordycepin had a protective effect against HIPI in mice by decreasing the release of inflammatory cytokines and inhibiting hepatocyte apoptosis and autophagy likely by inhibiting activation of the MAPK/NF-*κ*B signaling pathway.

## Figures and Tables

**Table 1:** Oligonucleotide sequences of primers used for qRT-PCR.

Gene	Forward (5′-3′)	Reverse (5′-3′)
*β*-Actin	GGCTGTATTCCCCTCCATCG	CCAGTTGGTAACAATGCCATGT
IL6	CTGCAAGAGACTTCCATCCAG	AGTGGTATAGACAGGTCTGTTGG
TNF-*α*	CAGGCGGTGCCTATGTCTC	CGATCACCCCGAAGTTCAGTAG
Bcl2	GCTACCGTCGTCGTGACTTCGC	CCCCACCGAACTCAAAGAAGG
Bax	AGACAGGGGCCTTTTTGCTAC	AATTCGCCGGAGACACTCG
Beclin-1	ATGGAGGGGTCTAAGGCGTC	TGGGCTGTGGTAAGTAATGGA
LC3	GACCGCTGTAAGGAGGTGC	AGAAGCCGAAGGTTTCTTGGG
NF-*κ*B	ATGGCAGACGATGATCCCTAC	CGGATCGAAATCCCCTCTGTT

## Data Availability

The data used to support the findings of this study are available from the corresponding authors upon request.
